# Investigating the impact of pedoclimatic conditions on the oenological performance of two red cultivars grown throughout southern Italy

**DOI:** 10.3389/fpls.2023.1250208

**Published:** 2023-09-15

**Authors:** Massimo Iorizzo, Angelo Sicilia, Elisabetta Nicolosi, Martino Forino, Luigi Picariello, Angela Roberta Lo Piero, Andrea Vitale, Eugenia Monaco, Filippo Ferlito, Mariantonietta Succi, Patrizio Tremonte, Angelita Gambuti, Clizia Villano, Antonello Bonfante, Riccardo Aversano, Raffaele Coppola

**Affiliations:** ^1^ Department of Agricultural, Environmental and Food Sciences, University of Molise, Campobasso, Italy; ^2^ Department of Agriculture, Food and Environment, University of Catania, Catania, Italy; ^3^ Department of Agricultural Sciences, Division of Grape and Wine Sciences, University of Naples Federico II, Avellino, Italy; ^4^ Institute for Mediterranean Agricultural and Forestry Systems, National Reaserch Council, Portici, Italy; ^5^ Council for Agricultural Research and Economics, Research Centre for Olive, Fruit and Citrus Crops, Acireale, Italy

**Keywords:** phenotypic plasticity, grape, soil, metabolites, microbiome, gene expression

## Abstract

The cultivated grapevine, *Vitis vinifera* subsp. *vinifera*, possesses a rich biodiversity with numerous varieties. Each variety adapts differently to varying pedoclimatic conditions, which greatly influence the terroir expression of wine regions. These conditions impact vine growth, physiology, and berry composition, ultimately shaping the unique characteristics and typicity of the wines produced. Nowadays, the potential of the different adaptation capacities of grape varieties has not yet been thoroughly investigated. We addressed this issue by studying two grape varieties, Aglianico and Cabernet Sauvignon, in two different pedoclimatic conditions of Southern Italy. We evaluated and compared the effect of different pedoclimatic conditions on plant physiology, the microbial quality of grapes using Next-Generation Sequencing (NGS) technology, the expression trends of key genes in ripe berries and the concentration of phenolic compounds in grapes and wines by HPLC-MS, HPLC-DAD, NMR and spectrophotometric analyses. Metabolomic and microbiome data were integrated with quantitative gene expression analyses to examine varietal differences and plasticity of genes involved in important oenological pathways. The data collected showed that the phenotypic response of studied grapes in terms of vigor, production, and fruit quality is strongly influenced by the pedoclimatic conditions and, in particular, by soil physical properties. Furthermore, Aglianico grape variety was more influenced than the Cabernet Sauvignon by environmental conditions. In conclusion, the obtained findings not only reinforce the terroir concept and our comprehension of grape’s ability to adapt to climate variations but can also have implications for the future usage of grape genetic resources.

## 1 Introduction

Among high-income crops, grapevine (*Vitis vinifera* subsp. *vinifera*) is considered one of the most challenged by climate change (Jones and Webb, 2010; Goode, 2012). In the Mediterranean area, a decrease in rainfall associated with an increase in temperature is expected (IPCC, 2022), leading to a shifting of suitable areas able to satisfy the specific thermal request of grapevine cultivars and to a marked effect on soil water availability. The combination of stressor conditions, and the expected water scarcity, will directly affect the grape and wine quality (Duchêne, 2016; Bonfante et al., 2017; Bonfante et al., 2018). Nevertheless, cultivated grapes possess a specific biological capability known as phenotypic plasticity, allowing them to adapt to climate changes; this adaptation is attributed to a comprehensive reorganization of the entire genome. Such reorganization often involves changes at the genomic, epigenetic, transcriptional, and network levels and is triggered by environmental factors. It is best illustrated by the concept of “terroir”, which encompasses the combined influences of varietal attributes, climate, soil conditions, winemaking practices, and the multitude of interactions among them (Bonfante and Brillante, 2022). Previous investigations showed a high level of differentiation among vineyards based on the varieties cultivated or by their area of provenance (Anesi et al., 2015; Paim Pinto et al., 2016; Xie et al., 2017; Dal Santo et al., 2018; Di Vaio et al., 2020). Concerning the varieties, a differentiation can be considered between international and indigenous ones. The former is more uniform and, thus, more resilient to environmental changes than the latter, indeed, international varieties tend to take over indigenous ones across the globe (Wolkowich et al., 2018). This knowledge gap needs to be filled to strengthen solutions towards declining grape diversity richness worldwide. Taken as a whole, the theme of phenotypic plasticity is complex, and researchers did not arrive at the same conclusions, with results sometimes controversial. This is likely linked to the fact that most grapevine studies focused on a single or a few components of the environment and plant responses. For example, Perin et al. (2020) and Vannozzi et al. (2023) observed genotype-dependent responses to soil factors in grapes. They highlighted the importance of specific soil properties, in influencing the metabolite profile of berry skin and found out that Corvina had higher biochemical plasticity compared to Glera, while Glera had higher transcriptional plasticity with respect to Corvina.

In recent years, few studies investigated the effects of various pedoclimatic conditions on berry and wine quality (Bonfante et al., 2018; Dinu et al., 2021; Rodrigues et al., 2023), while Nicolosi et al. (2022) have compared the phenotypic plasticity of indigenous grape varieties over international ones. In this work, we aimed to understand how pedoclimatic conditions can influence berries and wine characteristics, transcriptional regulation and the berries epiphytic fungal community of an international (Cabernet Sauvignon) and an indigenous (Aglianico) red grape variety along a latitudinal climatic gradient.

## 2 Materials and methods

### 2.1 Study areas

The study areas are in two hilly environments of southern Italy at different latitudes and elevations, Molise (41° 42’; 606 m a.s.l.), and Sicily (37° 40’; 400-700 m a.s.l.), in farms oriented to the production of high-quality wines. The parent material of soil is different between the experimental sites, clayey marls in Molise, and volcanic material in Sicily that led to the development of Vertisols and Andosols, respectively. The studied grapevines were Aglianico (AGL) and Cabernet Sauvignon (CS) cultivars planted between 2008 and 2010 on 140 Ruggieri rootstocks. In Molise, vines were planted in north-south rows, trained according to espalier Guyot, placed in a flat area. In Sicily, both vineyards were bush trained at 0.5 m, with two to six main branches (each branch was spur-pruned to one spur, with two buds per spur), and placed in anthropic terraces.

### 2.2 Pedoclimatic characterization and weather monitoring

In each experimental site, the representative soil profile has been described and analyzed. Soil profiles were described according to IUSS (IUSS Working Group WRB, 2014). Chemical analyses were performed according to the official methods of the Italian Ministry of Agriculture and Forestry (Colombo and Miano, 2015). Disturbed and undisturbed soil samples (volume 750 cm^3^) were collected from each soil horizon to determine chemical and physical soil characteristics. The hydraulic properties were determined in the laboratory according to the procedures reported by Basile et al. (2012) and Bonfante et al. (2010). Climatic characterization of each site was realized on the reference climate dataset 1971-2005 of Italy (spatial resolution about 8 km) realized and optimized on regional high-resolution observational data sets by Bucchignani et al. (2015). This choice has been necessary because there was no available local long-weather dataset reliable near the sites. Weather information (temperature, rainfall, solar radiations, wind speed, air humidity, etc.) was collected using an *in-situ* weather station (netsense.com) placed in each experimental vineyard within the canopy. Daily reference evapotranspiration (ET_0_) was estimated according to the equation of Penman-Monteith equation (Monteith, 1965) and the bioclimatic index of Amerine and Winkler (A&W; Amerine and Winkler, 1944) applied to compare the different thermal regimes.

### 2.3 Berry sample collection

Berries were collected at the full ripening stage (BBCH stage 89, Lorenz et al., 1995) during the 2020 growing season for biochemical, microbiological, and transcriptional analyses, all at the same time. Thirty clusters were harvested from different positions of the vineyard and from random positions on the plant to ensure the representation of the entire vineyard. At least ten berries were randomly selected from different parts of the cluster, avoiding those with visible damage and/or signs of pathogen infection, and pooled with berries from the other plants. For subsequent analyses, three independent pools (biological replicates) of 50 (for transcriptional analysis) or 100 (for biochemical and microbiological analyses) whole berries were selected, immediately frozen in liquid nitrogen, and stored at -80°C for subsequent analyses. For each analysis, four experimental samples were considered: Aglianico Molise (AM), Aglianico Sicily (AS), Cabernet Sauvignon Molise (CM), and Cabernet Sauvignon Sicily (CS).

### 2.4 Morpho-physiological measurements and maturity index calculation

For each cultivar/terroir combination and time of measurement, 21 fully exposed primary leaves, and 21 lateral leaves were collected. All the measurements have been conducted at the end of flowering, at the berry’s pea-sized stage, and at berry softening (vèraison). The total leaf area (TLA) was estimated through the measurement of leaf area (LA) of the main and lateral leaves realised in the lab with the area meter LI-3100 (Licor, Inc., Lincoln, Nebraska). Plant water status was determined in each experimental site using midday leaf water potential (LWP) measurement realised with a Schöelander pressure chamber (Soil Moisture Equipment Corp., Sta. Barbara, CA, USA; Scholander et al., 1965). Bunches were collected from 5 vines per treatment on each block for yield assessment. A sample of five bunches per treatment on each block was used to determine the bunch weight and both the number and weight of the berry. A 100-berry sample per experimental block was divided into three sub-samples, crushed with a manual press. The free-run juice was utilised to determine the total soluble solids (TSS) measured by a digital refractometer with temperature correction (RX-5000 Atago Co., Ltd., Bellevue, WA, USA). Must pH and titratable acidity (TA) were determined using an automatic titrator (Titrino model 798, Metrohm, Riverview, FL, USA) on 5.0 mL juice samples titrated against 0.1 M NaOH up to pH 8.2. TA was expressed as g/L of tartaric acid equivalents. The maturity index was estimated at harvest in 2020 (soluble solids -Brix° and pH), using the following formula (Lana et al., 2021):


$$\sqrt{pH}*{}^{\circ}Brix$$

### 2.5 Chemical analyses and spectrophotometric measurements of grape extracts and wines

Separation and extraction of berry components was carried out in duplicate simulating the maceration process necessary to produce red wines (Mattivi et al., 2002). Monomeric anthocyanins were measured according to the OIV Compendium of International Methods of Analysis of Wine and Musts (https://www.oiv.int/OIV-MA-AS315-11). Total flavanols were determined as vanillin-reactive flavans, according to Gambuti et al. (2015). Total phenolic compounds were dosed as iron-reactive phenolics (Harbertson, 2003). A large-scale extraction (300 g of fresh product) was carried out for each collected sample of red grapes. Destemmed grapes were crushed and extracted twice overnight Skin with 500 mL of H_2_O:EtOH 2:8 (v/v) mixture. The hydroethanolic extracts from each sample were concentrated under a vacuum and partitioned first against Ethyl Acetate (EtOAc) and then n-butanol 2-mL aliquots of each obtained extract were lyophilized and investigated by NMR. Likewise, 1-mL aliquots from each extract were lyophilized, solubilized in methanol, and subjected to MS analysis. NMR experiments were run on a Varian Unity Inova 700 spectrometer equipped with a 13C Enhanced HCN Cold Probe and by using a Shigemi 5 mm MR tubes. CD3OD (δH 3.31; δC 49.0) was selected as deuterated solvent. Standard Varian pulse sequences were employed for the respective classes of spectra. All NMR data reported in the text were derived from 1D 1H spectra and from 2D experiments, including COSY and TOCSY. Relative quantitation of each identified compound was conducted by selecting representative NMR signals and their areas determined by integration. For Mass Spectrometry analysis, a linear ion trap LTQ Orbitrap XL hybrid Fourier Transform MS (FTMS) instrument equipped with an ESI ION MAX source (Thermo-Fisher, Waltham, MA, USA) and coupled to an Agilent 1100 LC binary system was used. All the employed solvents were of laboratory grade. Following the NMR and High-Resolution MS data for the identified compounds: *Catechin:* HRESIMS *m/z* 291.0800 [M+H]^+^ corresponding to C_15_H_15_O_6_. *Epicatechin:* HRESIMS *m/z* 291.0857 [M+H]^+^ corresponding to C_15_H_15_O_6_. *Gallic acid:* HRESIMS *m/z* 169.0169 [M-H]^-^ corresponding to C_7_H_5_O_5_. *Syringic acid:* HRESIMS *m/z* 197.0501 [M-H]^-^ corresponding to C_9_H_9_O_5_. *Quercetin:* HRESIMS *m/z* 303.1858 [M+H]^+^ corresponding to C_15_H_11_O_7_. NMR data for the above compounds were consistent with those reported in the literature (Forino et al., 2019; Gambuti et al., 2020).

Analyses of monomeric anthocyanins were performed by an HPLC Shimadzu LC10 ADVP apparatus (Shimadzu Italy, Milan, Italy) equipped with an SCL-10AVP system controller, two LC-10ADVP pumps to create the needed solvent gradient, an SPD-M 10 AVP detector and an injection system full Rheodyne model 7725 (Rheodyne, Cotati, CA, USA). The HPLC solvents were the following: solvent A: water milli- Q (Sigma-Aldrich, Milan, Italy)/formic acid (Sigma- Aldrich ≥ 95%)/acetonitrile (Sigma-Aldrich≥ 99.9%) (87:10:3) v/v; solvent B: water/formic acid/acetonitrile (40:10:50) v/v. The gradient was: zero-time conditions 94% A and 6% B; after 15 min, the pumps were adjusted to 70% A and 30% B, at 30 min to 50% A and 50%B, at 35 min to 40% A and 60% B, at 41 min, end of the analysis, to 94% A and 6% B. 5 minutes re-equilibration time were applied before the subsequent analysis. The column used for the analyses was a water spherisorb column (C 18, Silica particle substrate, ODS2 250 x 4.6 mm, 5 μm particles diameter, 80 Å pore size) with a pre-column was used. 50 µL of calibration standards or wine was injected into the column. The absorbance signals at 520 nm was detected. Detector sensitivity was 0.01 Absorbance units full scale (AUFS). All the samples were filtered through 0.45 µm Durapore membrane filters (Millipore-Ireland) into glass vials and immediately injected into the HPLC system. The calibration curve was obtained by injecting 5 solutions (in triplicate) containing increasing concentrations of malvidin-3-monoglucoside (Extrasynthese, Lyon, France).

### 2.6 Grapes fungal community analysis

Fungal community composition was analysed by a culture-independent approach using next-generation sequencing (NGS). The grape samples from every vineyard were collected in duplicate, immediately transported to the laboratory and processed. Total genomic DNA was extracted using the Stool DNA Isolation Kit (Norgen, Biotek Corp., Thorold, ON, Canada) according to the manufacturer’s instructions. The concentration and purity of the extracted nucleic acids were visualised and quantified, after agarose gel electrophoresis, by Nanodrop (NanoDropTM 2000/2000c; Thermo Fisher Scientific, Italy). DNA quantity was standardized to a concentration of 10 ng/μl. The ITS2 (internal transcribed spacer) region of the rRNA was amplified using primers 2024F and 2409R. Amplicons were sequenced using the Illumina MiSeq PE300 platform (Illumina, San Diego, CA, USA) at Biodiversa s.r.l. (Rovereto, Italy). Adapter sequences were removed using Cutadapt (Martin, 2011). Read quality was assessed using DADA2 (Callahan et al., 2016). The taxonomic references were assigned using trained OTUs at 99% from UNITE database version 8.2 (https://unite.ut.ee) within Qiime2 tools version 2020.2 (https://qiime2.org) (Bolyen et al., 2019). The raw data have been deposited in Mendeley Data with the accession number DOI: 10.17632/tp2rrdz8wp.2.

### 2.7 Gene expression analysis in grapes

Total RNA was isolated from 40 mg of ground whole berries, excluding seeds. The Spectrum™ Plant Total RNA kit (Sigma-Aldrich, St. Louis, MO, USA) was used following the manufacturer’s protocol with some modifications (400 mg of fresh tissue powder and an additional Column Wash step). The quantity and quality of the isolated RNA was measured using the NanoDrop ND-1000 spectrophotometer (Thermo Scientific, Wilmington, DE, USA) and Qubit 2.0 fluorometer (Life Technologies, Carlsbad, CA). For cDNA synthesis, 100 ng of each RNA sample was reverse transcribed using the SuperScript® III cDNA Synthesis Kit (Life Technologies), following the manufacturer’s protocol. Expression analysis was conducted by Real-Time quantitative PCR (RT-qPCR) using an SYBR Green method on a 7900HT Fast Real-Time PCR System (Applied Biosystems, Foster City, CA, USA). Each 15 μl PCR reaction contained 330 nM of each primer, 2 μl of 5-fold diluted cDNA and 7.5 μl of SYBR Green Mix (Applied Biosystems, Foster City, CA, USA). The SDS 2.3 and RQ Manager 1.2 software (both Applied Biosystems, Foster City, CA, USA) were used for data elaboration. Primer pairs used in the quantitative analysis were divided into five groups based on their roles: glutathione S-transferase as precursors of volatile thiols, terpene synthase enzymes for terpenoid biosynthesis, O-methyltransferases for the biosynthesis of methoxypyrazines, anthocyanin biosynthetic pathway genes and peroxidase for anthocyanin degradation (Sparvoli et al., 1994; Kobayashi et al., 2002; Dunlevy et al., 2010; Kobayashi et al., 2011; Battilana et al., 2017; Sicilia et al., 2020; Ji et al., 2021; Maritim et al., 2021; Wang et al., 2021). The relative expression value was calculated through the ΔΔCt method using the Sicilian berries as calibrators (Livak and Schmittgen, 2001; Villano et al., 2016). Three biological and three technical replicates were considered.

### 2.8 Statistical analysis

To evaluate the environmental difference between the experimental sites, the well-known t-test and f-test methods were applied to the weather information collected in 2020: mean temperature, day-night temperature excursion, ET_0_, maximum wind, the Amerine and Winkler index (A&W), and rainfall. Quantitative grapevine data were compared using Tukey’s least significant differences procedure; all the variance resulted homogeneous. When the variances were not homogeneous, data were analyzed using Kruskall–Wallis test. When results of the Kruskal–Wallis test were significant (p< 0.05), the significance of between-group differences was determined by the Bonferroni–Dunn test (5% significance level). These analyses were performed using XLSTAT (version 2013.6.04; Addinsoft, Paris, France). All the data are expressed as means ± standard deviation of four replicates (two experimental replicates x two analytical replicates).

## 3 Results

### 3.1 Pedological and climatic characterization

**Figure 1:**
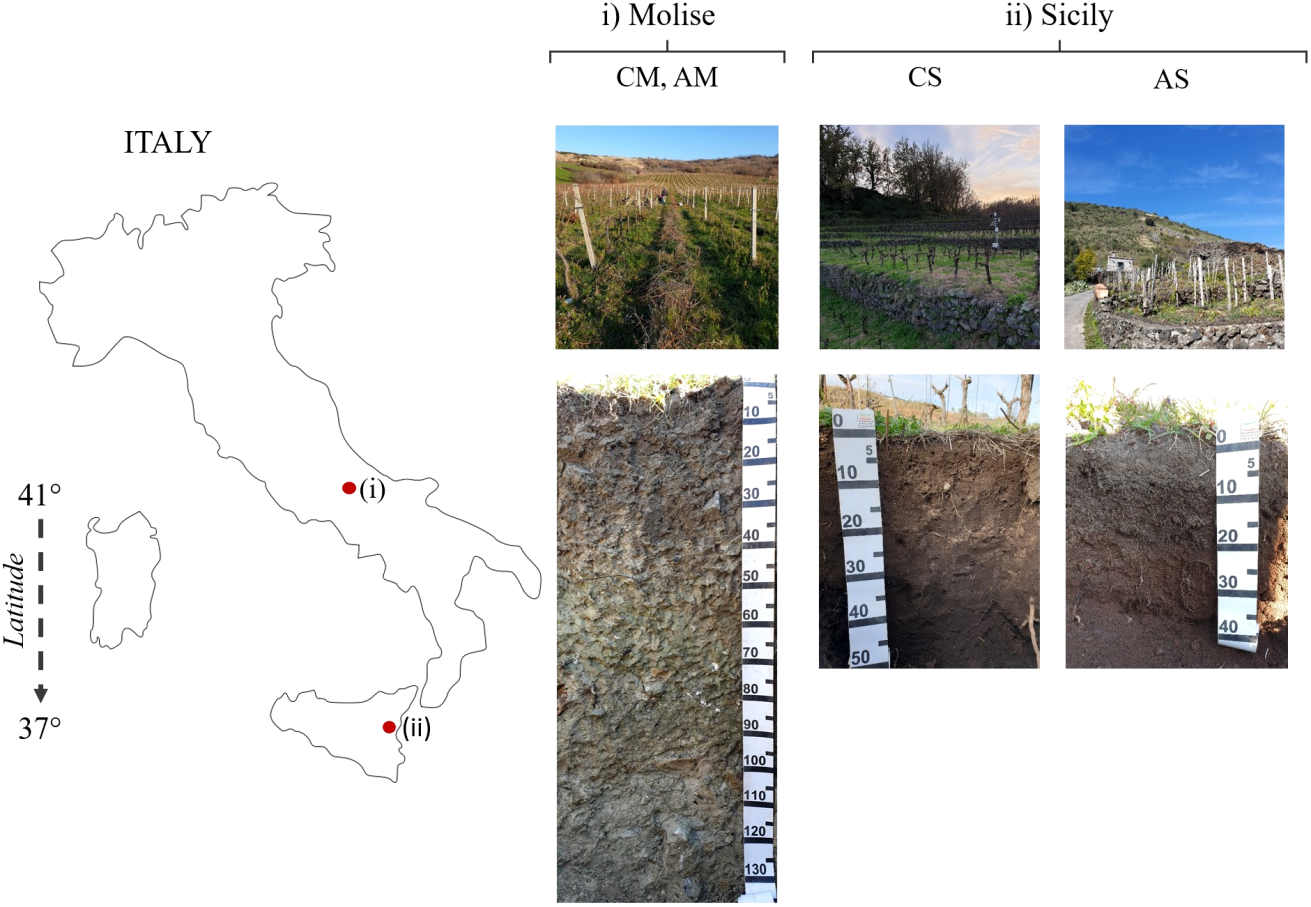
Experimental sites and representative soils.

In the two experimental sites, different soils were identified and described as representative of selected vineyards (**Figure 1** and **Table 1**). In particular, a deep clay soil in Molise site (Gleyic Calcic Vertisols) for both AGL and CS, and two shallow volcanic sandy soils (Vitric Andosols) in the Sicilian site. The available water capacity (AWC) of the first 100 cm differed among the soils, with the highest value expressed in Molise (205 mm) and the lowest in Sicily (127-132 mm). The topsoil layer cation exchange capacity (CEC) was high in both sites except for the Sicilian AGL vineyard. This data agrees with the young nature of the latter soil, less evolved compared to the others (96.1% of volcanic sand), and less enriched in organic matter compared to the Sicilian CS soil. Moreover, a rising of water from a suspended groundwater table was found in the Molise site.

**Table 1:** Soil properties of experimental sites.

Site/soil	Soil horizon and thickness (cm)		Particle size fraction			OM	Total Nitrogen	Total Limestone	C/N	pH	CEC	ECe	AWC(0-100 cm)
			Clay	Silty	Sand								
			(g 100 g^-1^)								(meq 100g^-1^)	(dS m^-1^)	mm
Molise (AM, CM)/ Gleyic Calcic Vertisols	Ap1	0-5	42,9	19,0	38,1	4,1	0,25	13,1	9,3	7,9	33,9	0,23	205
	Ap2	5-30/45	47,3	21,5	31,2	3,1	0,20	13,5	8,9	8,3	32,5	0,14	
	Bwk	30/45-80	55,2	18,1	26,7	0,8	0,07	22,5	6,5	8,5	29,7	0,12	
	BC	80+	39,1	31,6	29,3	1,2	0,06	17,3	11,6	8,5	27,9	0,11	
Sicily (CS)/ Vitric Andosols	Ap1	0-20	2,2	4,9	92,9	4,7	0,25	0,0	10,8	6,9	13,5	0,05	132
	Ap2	20-45	1,8	6,3	91,9	3,8	0,20	0,0	11,2	7,1	14,5	0,03	
	Bw	45+	0,7	7,8	91,5	4,4	0,24	0,0	10,6	7,2	20,0	0,03	
Sicily (AS)/ Vitric Andosols	Ap	0-20	0,5	3,4	96,1	2,3	0,14	0,0	9,3	7,7	7,9	0,05	127
	Bw	20-40+	1,1	1,8	97,1	1,4	0,07	0,0	12,0	7,5	4,5	0,03	

### 3.2 Weather data and climatic characteristics of experimental sites

**Figure 2:**
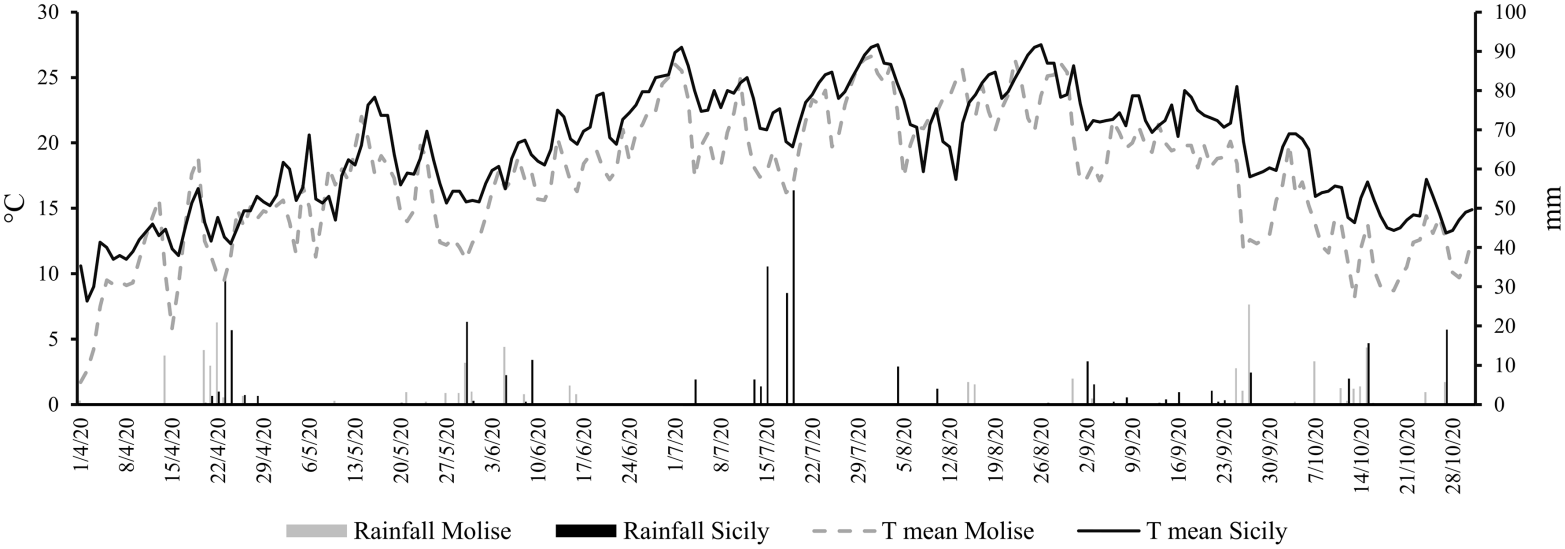
Daily mean temperatures (T mean) and daily rainfall (mm) across the selected vineyards in the Molise and Sicily sites from 1 April 2020 to 31 October 2020.

**Figure 3:**
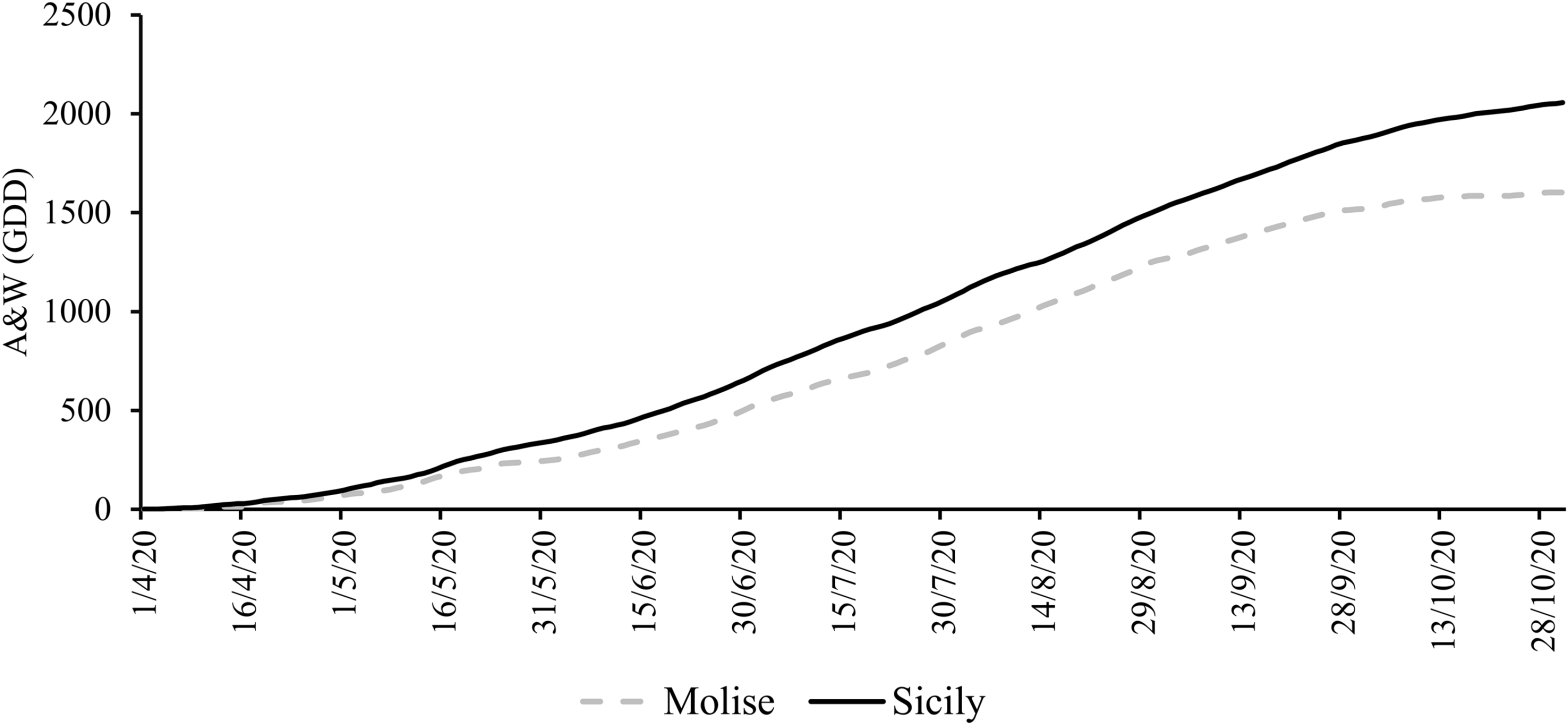
Cumulated Amerine and Winkler index (Growing Degree Days - GDD) trend in the experimental sites (1 April - 31 October 2020).

The results of climate analysis (RC 1971-2005 dataset) explained the effects of latitude in a Mediterranean climate (cold winters and mild summers), with the high latitude site (Molise) being the coldest compared to the Sicilian site (low latitude). Looking at the grapevine growing season (April-October 2020), the mean temperature was about 7.6% higher in Sicilian than in the Molise site. The behavior of the A&W index reflected the temperature differences, with a gap of 15% between Sicily and Molise. The ET_0_ for the two sites was quite similar, with a small difference of 9%, while the cumulative rainfall was lower for Sicily compared to Molise (average difference of -8%). Daily mean temperatures and rainfall collected in each experimental site during the monitored growing season (April-October) are reported in **Figure 2**. The mean temperature over the selected time was 17.2°C ( ± 0.5) for the Molise site, and 19.6°C ( ± 0.4) for the Sicilian one, and the ET_0_ was 748.4 mm ( ± 1.4) and 714.3 mm ( ± 1.8). The accumulated rainfall was 219.5 mm and 333.4 mm for Molise and Sicily, respectively. The thermal regime showed a higher value of the A&W index in Sicily (2056 GDD compared to 1600 GDD of Molise) with a different trend in the accumulation of GDD during the growing season (**Figure 3**). Statistical analysis of weather conditions among the experimental sites showed a significant difference between the two sites with a p< 0.05 (p mean temperature = 9,6E-27, p day-night temperature excursion = 0,0036, p ET_0_ = 0,0325, p maximum wind = 1,96E-51 and p A&W index = 4,29E-55), with an average difference of mean temperature = 2,4 C°, day-night temperature excursion = -0,9 C°, ET_0_ = -0,2 mm, maximum wind = -2,3 ms^-1^ and A&W index = 191,4 GDD. Regarding rainfall, there is no statistical difference between the two sites, but from the biological point of view, the difference of 100 mm over the growing season between Molise and Sicily can be considered significant in terms of the effect on plant responses.

### 3.3 Morphological measurements

During early summer, midday LWP was about less than -0.8 MPa in both environments, which is considered the negative threshold for well-irrigated vines (Olivo et al., 2009; Intrigliolo et al., 2012). Indeed, the ψ ≤ -1.0 MPa recorded from midsummer to the end of the season, are considered drought-like conditions occurring from the fruit set throughout the ripening stage. During the latter period in Sicily, the registered values of -1,79 MPa for Aglianico and -1.73 MPa for Cabernet Sauvignon were significantly below -1.2 MPa or even that is considered a condition of severe stress (Nicolosi et al., 2022). The LWP was measured at the end of flowering, at berry’s pea-sized stage and at berry softening. The values were significantly different between the first and third stages for Aglianico. LWP values were -1.31MPa (AS) and -1.79 MPa (CS) in Sicily, whereas in Molise they were higher. During the intermediate stage (berry’s pea-sized stage) no differences were detected. As regards Cabernet Sauvignon, the most stressed vines were observed in Molise during both the end of flowering and berry’s pea-sized stage (ψ= -1,12 MPa *vs* -0,95 MPa, and ψ= 1,32 MPa vs -1,15 MPa, respectively). At berry softening the most stressed vines were in Sicily (-1,73 MPa *vs* -1,17 MPa) (Nicolosi et al., 2022).

The total leaf area per vine (TLA) is reported in **Table 2**. For both cultivars and each phenological stage, the Molise site showed significantly higher values than the Sicilian site. However, in the latter, for Cabernet Sauvignon the TLA slightly increased during the growing season, while in Molise because of the shoot topping (midsummer) a decrease was recorded. For Cabernet Sauvignon, the above-mentioned decrease was observed at the berry softening stage due to the manual late basal leaf removal. The main and lateral shoot behaviour influenced the TLA. In fact, for Aglianico in Sicily, the TLA/main shoot ratio increased during the experimental period (from 0.23 m^2^ to 0.34 m^2^), whereas in Molise, a decrease in TLA/main shoot ratio was registered between the end of flowering stage and the berry pea-size stage. Concerning the lateral shoots in Molise, a substantial increase occurred at the berry softening stage. For Cabernet Sauvignon, significant differences between the two experimental sites were observed at the end of flowering and at the softening of berries, both for main and lateral shoots. Regarding the leaf area index, a significant difference was recorded at the berry pea-size stage for Aglianico and Cabernet Sauvignon and the highest value was observed in Sicily. On the contrary, the leaf area index of Aglianico in Molise showed a significant increase at the berry softening stage.

**Table 2:** Vegetative behavior in each of two cultivars grown in two different sites.

Experimental vineyard	Total leaf area/vine (m^2^)			Total leaf area/main shoot (m^2^)			Total leaf area/lateral shoot (m^2^)			Leaf area index		
	End of flowering	Berries pea-sized	Softening of berries	End of flowering	Berries pea-sized	Softening of berries	End of flowering	Berries pea-sized	Softening of berries	End of flowering	Berries pea-sized	Softening of berries
AS	1.26±0.47^b^	1.70±0.52^b^	1.79±0.52^b^	0.23±0.06^b^	0.31±0.10^a^	0.34±0.08^a^	0.04±0.02^b^	0.07±0.02^b^	0.05±0.02^b^	1.14±0.43^n.s.^	1.55±0.47^a^	1.63±0.47^b^
AM	4.54±0.66^a^	3.85±1.02^a^	7.12±2.38^a^	0.30±0.03^a^	0.24±0.05^b^	0.25±0.08^b^	0.07±0.02^a^	0.10±0.03^a^	0.36±0.11^a^	1.31±0.19^n.s.^	1.11±0.29^b^	2.05±0.68^a^
CS	1.83±0.47^b^	2.70±0.60^b^	1.51±0.37^b^	0.18±0.04^b^	0.26±0.06^n.s.^	0.15±0.04^b^	0.05±0.01^b^	0.07±0.02^n.s.^	0.04±0.01^b^	1.28±0.33^n.s.^	1.88±0.42^a^	1.06±0.26^n.s.^
CM	4.45±1.02^a^	3.38±0.94^a^	3.85±0.87^a^	0.29±0.04^a^	0.18±0.04^n.s.^	0.18±0.04^a^	0.08±0.01^a^	0.09±0.03^n.s.^	0.14±0.05^a^	1.28±0.29^n.s.^	0.97±0.27^b^	1.11±0.25^n.s.^

Measurements were recorded at the phenological stages, BBCH69 end of flowering, BBCH75 berries pea-sized, BBCH85 softening of berries. Significantly different mean values for each parameter are indicated by different letters (lowercase letter p ≤ 0.05, uppercase letter p ≤ 0.001, ± indicate one standard deviation) based on Tukey’s HSD test.

### 3.4 Berry and wine characteristics

**Figure 4:**
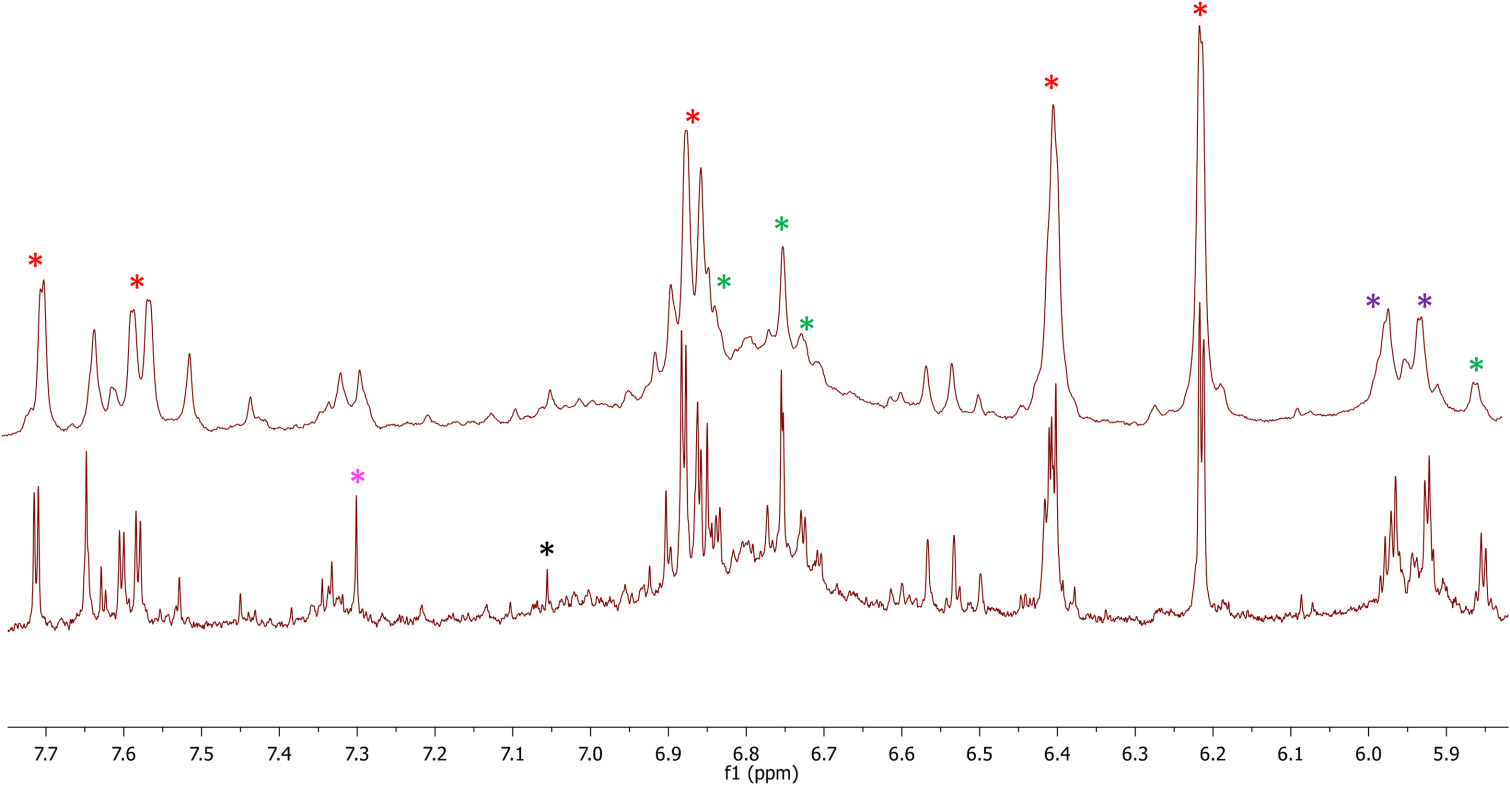
Enlargements of the 1H-NMR spectra of CM (bottom) and CS (top) containing resonances assigned to the identified polyphenols. Red asterisks indicate resonances of quercetin, green asterisks indicate resonances of (+)-catechin, purple asterisks indicate overlapped resonances of (+)-catechin and (-)-epicatechin, magenta asterisk indicates syringic acid, and black asterisk gallic acid.

The berries and wine characteristics are reported in **Table 3**. Aglianico and Cabernet Sauvignon had the highest yield, bunch weight, and berry number in Molise. As regards the berry weight, a significantly high value was detected in Molise for Aglianico, while the berry weight of Cabernet Sauvignon was similar in each region. The rachis weight was always higher in Sicily than in the Molise site. The technological maturity of grapes was evaluated by determining the content of soluble solids, titratable acidity, their ratio, and the pH in 2020. The main classes of phenolic compounds extractable from skins and seeds were also determined (**Table 3**). Total iron reactive phenolics, total flavanols and anthocyanins are higher in samples grown in Molise, while the content of flavanols strongly differs Aglianico from Cabernet. Data on alcohol content of wines were correlated to the content of sugars in grapes (**Table 3**). The lower values of titratable acidity of all wines with respect to respective grapes are expected considering tartaric precipitation during fermentation and post-fermentative aging of wines. Comparing data on total phenolics in grapes and wines (**Table 3**) in AS and AM phenolics were transferred from grapes to wine during winemaking but, especially for AM, a slight loss, probably due to adsorption on grape marcs, was detected. In contrast, in CS higher contents of phenolics compounds in wines with respect to berries were observed. This is easily explained since the method used to determine total phenolic compounds involved the reaction with iron and, thus, it is likely that during winemaking, for this grape variety, the formation of new phenolic structures highly reactive towards iron occurred. This is not surprising given the high reactivity of phenolic compounds among themselves and with fermentation metabolites such as acetaldehyde during winemaking. The lower values of total flavanols and anthocyanins detected in wines with respect to grapes is due to the great number of reactions of oxidation, polymerization, and precipitation that native phenolic compounds underwent. To identify the major polyphenols occurring in the analyzed samples, an NMR-based investigation was conducted. To this aim, both Aglianico and Cabernet Sauvignon grapes were extracted and partitioned as reported in the experimental section. Polyphenols were accumulated in the EtOAc extracts. Gallic acid and syringic acid were identified as phenolic acids, (+)-catechin and (-)-epicatechin and detected as the most abundant flavan-3-ols derivatives, while quercetin emerged as the most abundant flavonol compound. In **Figure 4**, ^1^H-NMR spectra of CM and CS EtOAc extracts display all the above proton resonances attributed to the identified polyphenols. Typical NMR signals attributable to hydroxycinnamic acids were identified, but due to their low intensities and occurrence in quite crowded spectral regions, unambiguous identification of specific molecules was prevented. It is to be underlined that anthocyanins were not analyzed by either NMR and MS techniques, as they were investigated by means of DAD-HPLC and spectrophotometric analyses, as reported above. All the above NMR-based structural hypotheses were corroborated by High Resolution Mass Spectrometry analysis (See Materials and methods).

**Table 3:** Yield, berry and wine characteristics of each experimental site and cultivar.

Parameter		Experimental site			
		AS	AM	CS	CM
Morphological information	Yield/vine (Kg)	0.24±1.07^B^	6.80±1.88^A^	2.45±0.43^B^	4.84±0.95^A^
	Bunch weight (g)	190.00±15.41^B^	558.60±161.50^A^	258.40±25.38^b^	352.60±74.82^a^
	Bunch length (cm)	16.00±1.87^n.s.^	16.80±0.84^n.s.^	17.60±1.52^b^	20.60±2.07^a^
	Berry number	79.80±7.66^B^	180.50±36.70^A^	125.80±19.23^b^	169.20±35.97^a^
	Berry weight (g)	2.15±0.03^b^	2.64±0.22^a^	1.90±0.12^n.s.^	1.97±0.17^n.s.^
	Rachis weight (g)	18.46±1.50^a^	16.80±1.64^b^	21.20± 1.64^a^	18.60 ±1.82^b^
Berry characteristics information	Soluble soilds (°Brix)	21.06±0.21^n.s.^	21.59±0.63^n.s.^	19.91±0.04^B^	22.33±0.89^A^
	Titratable acidity (g l^-1^)	5.32±0.37^B^	10.36±0.30^A^	5.14±0.11^B^	9.84±0.46^A^
	pH	3.55±0.03^n.s.^	3.48±0.21^n.s.^	3.61±0.14^b^	4.01±0.35^a^
	Maturity index	39.69±0.38^n.s.^	40.28±2.18^n.s.^	37.84±0.80^B^	44.64±1.53^A^
	Total Phenolics (mg kg^-1^)	1263.12±88.01^c^	1661.50±108.20^a^	1291.44±18.70^c^	1498.98±38.20^b^
	Total Flavanols (mg kg^-1^)	1554.50±58.10^b^	1977.63±87.40^a^	1066.41±75.27^d^	1339.28±95.60^c^
	Anthocyanins (mg kg^-1^)	788.12±35.45^c^	1248.04±26.20^a^	895.62±43.40^b^	1294.94±48.70^a^
Wine characteristics	Alcohol (%vV^-1^)	11.65±0.10^b^	12.50±0.20^a^	12.15±0.04^ab^	12.20±0.16^a^
	Titratable acidity (g l^-1^)	4.25±0.01^c^	7.17±0.08^a^	4.91±0.02^b^	5.30±0.26^b^
	pH	4.10±0.02^a^	3.32±0.04^a^	3.71±0.01^b^	3.71±0.05^b^
	Total Phenolics (mg kg^-1^)	956.92±119.50^a^	1879.74±39.60^a^	1635.36±147.0^a^	2465,95±100.30^a^
	Total Flavanols (mg kg^-1^)	282.08±30.59^bc^	527.71±106.01^a^	289.16±25.20^b^	188.75±28.90^c^
	Anthocyanins (mg kg^-1^)	128.49±13.30^b^	208.38±44.36^a^	134.53±30.84^b^	166.7±32.70^ab^

Significantly different mean values for each parameter and year are indicated by different letters (lowercase letter p ≤ 0.05, uppercase letter p ≤ 0.001, ± indicate one standard deviation) based on Tukey’s HSD test.

### 3.5 Characterization of fungal community

**Figure 5:**
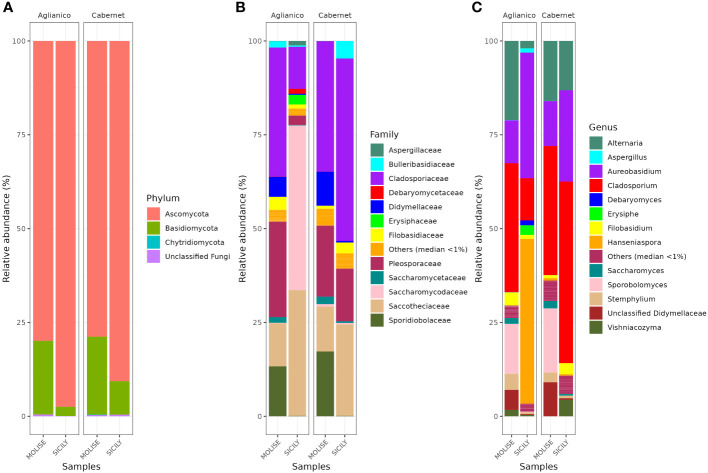
Relative abundance of grape fungal community at phylum **(A)**, family **(B)** and genus **(C)** level detected by NGS, Next Generation Sequencing.

The data on the fungal community present in the harvested grapes is shown graphically in **Figure 5**. Overall, at harvest time the fungal populations, present in the vineyards of the examined sites, at the phylum level are very similar (**Figure 5A**). *Ascomycota* was the most abundant phylum followed by *Basidiomycota*. Other phylums such as *Chytridiomycota* are present only in low abundance. The analysis of the fungal community by high-throughput sequencing allowed to identify a total of 13 families and 12 genera. At the family level (**Figure 5B**), mean values of relative abundance revealed that the population was mainly represented by the *Cladosporiaceae* family with relative percentages between 11.2 (AS) and 48.6% (CS). The *Saccotheciaceae* family was widely present on the grapes analyzed with relative percentages between 11.4% (AM) and 33.5% (AS). *Saccharomycodaceae* was among the predominant family on AS grapes with a relative abundance of 43.9% while on the other grapes sampled this family was little detected. Widespread presence of *Pleosporaceae* with relative abundance between 2.5% (AS) and 18.9% (CM). At the genus level (**Figure 5C**), the main contaminants of grapes were fungi belonging to the genera *Cladosporium, Aureobasidium, Alternaria and Sporobolomyces*. A significant presence of the genus *Hanseniaspora* was recorded only on AS grapes (43.9%). The diffuse presence of *Cladosporium* was recorded on all the grapes analyzed, with relative abundances between 11.2% (AS) and 48.4% (CS).

### 3.6 Transcriptional analysis of Aglianico and Cabernet Sauvignon berries

**Figure 6:**
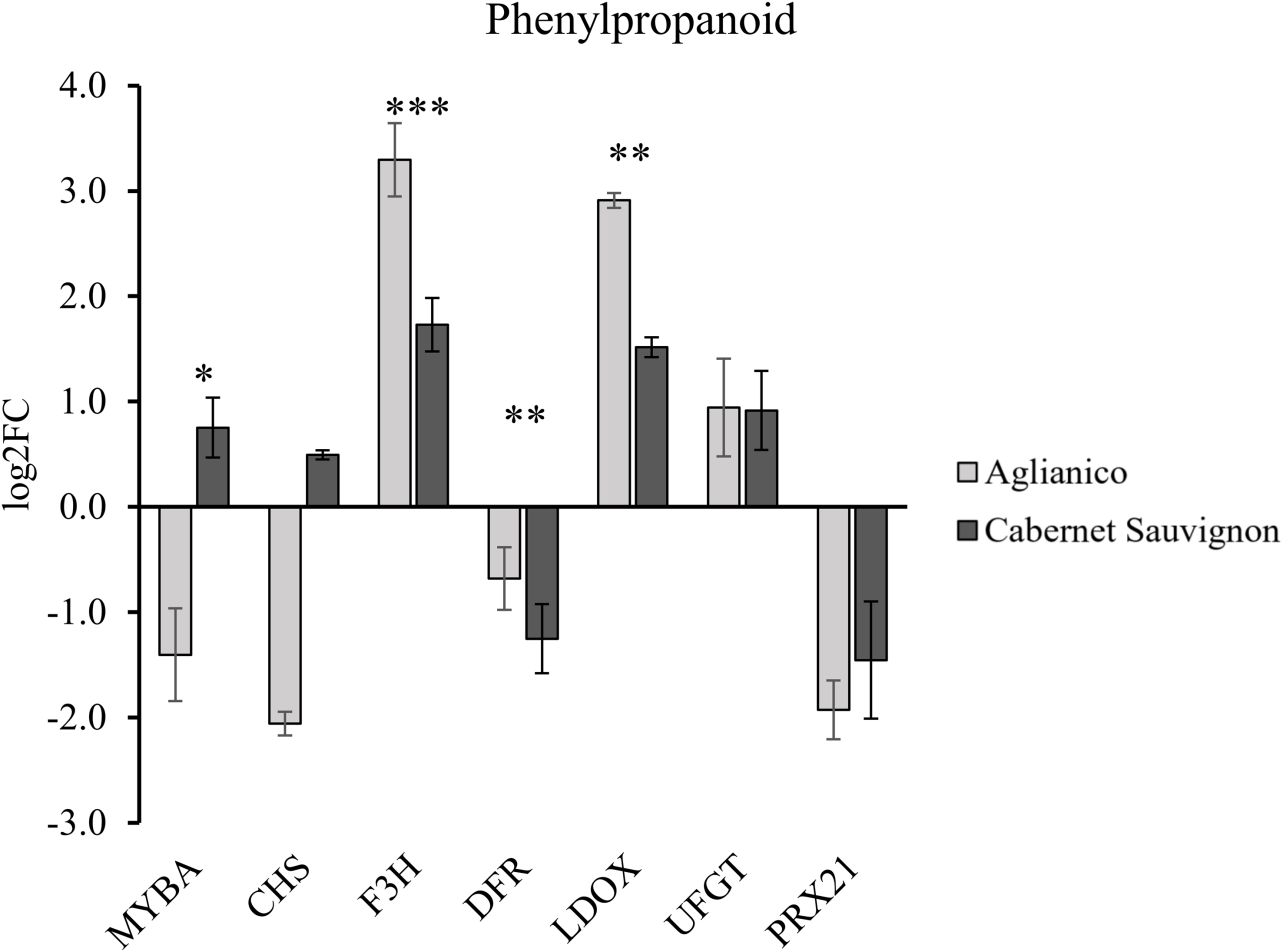
Transcript abundance of genes involved in phenylpropanoid (MYBA, CHS, F3H, DFR, LDOX, UFGT, GST, and PRX21) metabolism. The values are expressed in log2 of Fold Change and are normalized on Sicilian samples. Statistically significant differences are asterisked (* p ≤ 0.05, ** p ≤ 0.01, *** p ≤ 0.001).

The transcript abundance of representative key genes of phenylpropanoid metabolism is shown in **Figure 6** as log_2_ of the fold change of the samples from Molise normalized to the samples from Sicily. Overall, both varieties showed an overexpression of F3H, LDOX, and UFGT, while DFR and PRX21 were downexpressed. MYBA and CHS were overexpressed in Cabernet Sauvignon and downexpressed in Aglianico. As regards volatile thiol, methoxypyrazine, and terpenoid metabolism in Molise we observed, with respect to Sicilian grapes, an overexpression of *GST2*, *GST3* and *GGT* for volatile thiols, *OMT1* and *OMT2* for methoxypyrazine, and *TPS56* for terpenoids, in both Aglianico and Cabernet Sauvignon. On the counterpart, *OMT3* showed downregulation in both varieties (**Figure S1**).

## 4 Discussion

The phenotypic plasticity of grape varieties is heavily influenced by the environmental conditions they inhabit, affecting their vigor, production, and fruit quality. Although grapevine plasticity and viticulture practices may provide advantages related to the adaptation of a cultivar to specific growing conditions, it may also cause irregular ripening (Selvaraj et al., 1994) and large inter-seasonal fluctuations (Clingeleffer, 2010), which are undesirable characteristics for wine making (Keller, 2010). In this study we tested how the phenotypic plasticity of two red berries grapes, Aglianico and Cabernet Sauvignon, can be influenced by the pedoclimatic conditions in terms of berries and wine characteristics, transcriptional regulation, and epiphytic fungal community. Overall, we found that both varieties were significantly influenced by the specific pedoclimatic conditions in Molise and Sicily regarding vegetative and productive responses. The sampling sites had a different capacity in soil function expression due to substantial differences in terms of chemical and physical characteristics. This function depends mainly on soil hydraulic properties that can drive grapevine responses and influence the quality of the grapes (Bonfante et al., 2017; Brillante et al., 2017; Brillante et al., 2018). In our experimental sites, the hydraulic properties were higher in Molise compared to Sicily as well as the weather variables affecting the soil water balance with about 9% ET_0_ in average difference. Such environmental differences between the sites led to different physiological and productive behaviors in both varieties. They can be considered responsible for the growth rate reductions of Aglianico in Sicily from the pea-size berries to the softening of berries stage. Indeed, a depressive effect of drought on shoot elongation and leaf emergence was observed, in agreement with negative water potential found. In contrast, in Molise, the Aglianico growth rate was continuous (mainly for lateral shoots), determining a very long vegetative and reproductive cycle with a high canopy efficiency. By contrast, Cabernet Sauvignon registered a less negative water potential value despite reduction of its leaf area. Furthermore, in Sicily, the growth of both the main and lateral shoots stopped, and the basal leaves lost their functional capabilities. It is crucial to emphasize that the observed discrepancies among sites regarding Cabernet Sauvignon are likely attributed more to the variations in training systems rather than the pedoclimatic conditions. Specifically, the reductions in leaf area index observed in Molise during mid-summer resulted from vine hedging carried out during that period. Consequently, the observed behavior could be due to a better adaptability of this cultivar, which adapted its phenotype in response to various restrictive growth conditions. Concerning Aglianico, the crop load was significantly impacted by the prevailing environmental conditions, leading to notably lower yields in Sicily due to an extended period of severe drought, compared to the Molise site, where abundant water availability fostered vigorous vegetative growth. Consequently, the genotype-environment interaction for Aglianico exhibited its strongest effect during the berry ripening phase. The NMR-based analysis of grape extracts highlighted interesting differences between Molise and Sicily regarding the relative abundance of flavonols, especially in Cabernet Sauvignon. The polyphenolic profile of this variety turned out to be qualitatively superimposable by confronting the relative abundance of quercetin with that of (+)-catechin. Quercetin was more concentrated than (+)-catechin in Sicily than Molise. Furthermore, flavonols concentration was higher in Sicily than in Molise. It might not be surprising since flavonols act in grapes as photo-protectors (Price et al., 1995; Gambuti et al., 2019; Gambuti et al., 2020), and in Sicily, plant tissues were more exposed to sunlight than in Molise. To understand whether this effect detected on grapes is determinant also for wine quality, the corresponding wines were obtained from each grape experimental sample with the same winemaking protocol and evaluated base parameters together with the content of total phenolics, flavonols, and anthocyanins. In regard to the Aglianico, unlike the Cabernet ones, our NMR-based investigation did not bring to light any differences among the identified polyphenols from either qualitative or quantitative standpoints. As already observed in Aglianico grapes (Forino et al., 2019), the content of (+)-catechin (83.1 ± 1.2 mg/L) and (-)-epicatechin (103.4 ± 1.8 mg/L) was comparable, whilst quercetin was not detected under our NMR experimental conditions. In contrast, our results on berries in terms of titratable acidity and pH showed that Aglianico is more sensitive to the environment compared to Cabernet. This datum is relevant and should be confirmed in further years given the relevance of acidic profile for microbial contamination, oxidation risk, and sensory quality of wines. Differences observed in grapes for total flavanols of Cabernet between Molise and Sicily were not confirmed in wines, probably for the extractability of these compounds in winemaking and to the numerous reactions of polymerization and precipitation in which flavanols are involved and that occur just after grape crushing until wine bottling (Forino et al., 2020). Regarding the role of variety on phenolic composition, the amount of flavanol in Aglianico wines was higher than in Cabernet Sauvignon, as already detected for these grape cultivars (Gambuti et al., 2006). For both cultivars, passing from the higher latitude (Molise) to the lowest (Sicily), their content decreases. It is known that the biosynthesis of anthocyanins depends on UV radiation and thermal excursion. The higher the radiation and the thermal excursion, the higher the synthesis of anthocyanins. In contrast, high night temperatures determine the degradation of anthocyanins during berry ripening (Teixeira et al., 2013). Therefore, Sicily’s higher night temperature and lower thermal excursion can easily explain the significant difference between Molise and Sicily for both cultivars. In this sense, the most sensitive cultivar to latitude was the Aglianico, which doubled the total monomeric anthocyanins passing from Sicily to Molise. In contrast, previous studies on Pinot Noir grapevines sampled across a latitudinal gradient in Europe, from southern Spain to central Germany showed that total anthocyanins did not show any correlation with radiation variables (Del-Castillo-Alonso et al., 2016). This confirms the paramount importance of cultivars. The data on anthocyanins in wines also confirmed this dependence on latitude. We observed higher levels of anthocyanins in Cabernet Sauvignon compared to Aglianico. Specifically, when comparing the individual environments, grapes of both varieties cultivated in Molise showed a richer anthocyanin pigment content compared to the corresponding varieties grown in Sicily. This finding aligns with the expression analysis of biosynthetic genes, revealing higher expression levels of F3H, LDOX, GST2, GST3, and GGT in Molise. In Sicily, we recorded reduced anthocyanin accumulation along with low or insignificant levels of biosynthetic gene expression (DFR, UFGT) and a higher degradation rate. This could be attributed to the climatic conditions in Sicily, where heat and drought stressors coincide. Previous studies have shown that drought conditions up-regulate anthocyanin biosynthesis (Castellarin et al., 2007), while heat stress negatively affects anthocyanin concentrations in grapes (Mori et al., 2007; Movahed et al., 2016; Pastore et al., 2017). Water-restricted cultivation has been suggested as a potential countermeasure to mitigate the negative impact of excessive heat on anthocyanin levels, particularly during later growth stages like berry ripening (Scholasch and Rienth, 2019). Recent findings by Tan et al. (2023) also support this, showing the downregulation of anthocyanin biosynthesis genes during combined drought and heat stress in Cabernet Sauvignon. Among the other examined classes, OMT1 and OMT3 have been found to exhibit a significant correlation with the methoxypyrazine content (Zhang et al., 2022). Our study revealed the overexpression of OMT1 in Sicily and varying levels between Aglianico and Cabernet Sauvignon, supporting the hypothesis that methoxypyrazine accumulation in grapes is influenced by light exposure and genotype (Koch et al., 2010; Sanders et al., 2023; Villano et al., 2023). The observed variations in grape characteristics, including soluble solids content, acidity, and pH, highlight the intricate relationship between environmental conditions and grape quality. These factors not only impact the composition of the grapes but also have implications for their fermentation capability and the ultimate sensory attributes of the resulting wines. Indeed, the effect of environmental conditions on grapes’ fermentation capability can be expressed by the ratio between soluble solids content and titratable acidity, which helps to understand if grapes can give problems during fermentation and if they can give equilibrated wines from the gustative point of view. Values too high of this ratio indicate that the fermentation of these grapes is not easy, and several problems such as sluggish or arrest of fermentation, could happen, as well as the appearance of microbial contamination. In addition, the obtained wines cannot be equilibrated and show several off-flavours. In our case studies, this ratio next to 4, well confirms the strong link between grape quality for fermentation and environmental conditions, especially for the Aglianico grapevine. In addition to chemical characteristics of grapes, our results, obtained using NGS method, reinforce the concept that also the grape yeast community composition differs according to geographical location and this biodiversity is a factor that could contribute to regional wine characteristics (Bokulich et al., 2016). In recent years, the contribution of the “native” (non-inoculated) yeasts to improving regional typicality has been enhanced (Liu et al., 2019). In fact, the specific grape-associated microbiota can exert both positive and negative effects on the quality of wine before, during and after fermentation (Spano and Torriani, 2016). This connection between microbial biogeography and regional wine characteristics has been termed “microbial terroir”, a term that hypothesizes a relationship between grapevine microbiology and wine terroir (Gilbert et al., 2014; Morgan et al., 2017).

## 5 Conclusion

In conclusion, the phenotypic response of Aglianico and Cabernet Sauvignon grapevines, encompassing vigor, production, and fruit quality, is significantly influenced by the prevailing pedoclimatic conditions, particularly soil physical properties. These conditions play a crucial role in shaping grape acidity, notably through the accumulation of organic acids, while their impact on pH expression appears to be comparatively less significant. Furthermore, pedoclimatic factors influence the content of total monomeric anthocyanins in grapes and wines, reflecting their effect on the expression of grapevine genes involved in phenolic and aroma compound metabolism, as well as qualitative traits. The findings presented in this study strongly support the concept of terroir and provide valuable insights into the current adaptation of vineyards and terroirs to climate change. Moreover, these results underscore the importance of comprehending the genetic plasticity of vine cultivars in navigating such environmental shifts.

## Data availability statement

The original contributions presented in the study are publicly available. This data can be found here: https://data.mendeley.com/datasets/tp2rrdz8wp/2.

## Author contributions

RA, AP, AB and RC conceived and designed research. MI, AS, EN, MF, LP, AV, EM, FF, MS, PT, CV and AB conducted experiments. MI, AS, EN, MF, LP, RA, AP, AV, EM, FF, MS, PT, AG, CV and AB analyzed data. MI, AS, MF, RA, AP, FF, AG, CV and AB wrote the manuscript with comments and input from all authors. RA, AG, CV, AB and RC revised the manuscript. All authors contributed to the article and approved the submitted version.
