# Supplementary material for: Investigating the impact of pedoclimatic conditions on the oenological performance of two red cultivars grown throughout southern Italy
Source: Front Plant Sci. 2023 Sep 15;14:1250208. doi: 10.3389/fpls.2023.1250208 (PMC10540683; doi:10.3389/fpls.2023.1250208)
Supplement: Supplementary file 1 [file Image_1.pdf]

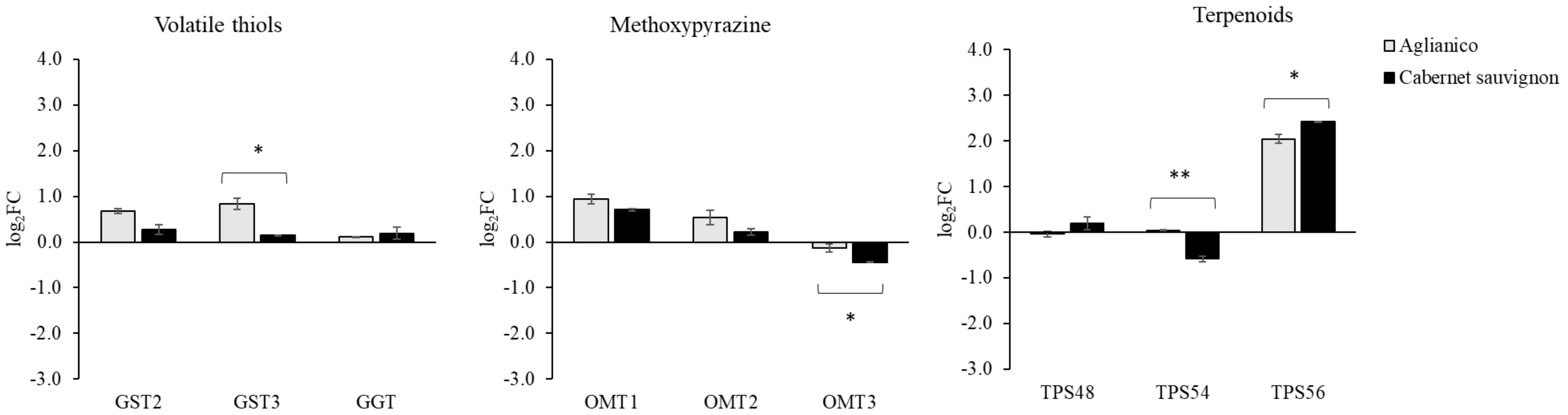

Figure S1 - Transcript abundance of genes involved in volatile thiols (GST2, GST3 and GGT), terpenoids (TPS48, TPS54, TPS56) and methoxypyrazine (OMT1, OMT2, OMT3). The values are expressed in log_2 of Fold Change and are normalized on Sicilia n samples. Statistically significant differences are asterisked (* $p \leq 0.05$, ** $p \leq 0.01$, *** $p \leq 0.001$, **** $p \leq 0.0001$)
